# The diagnostic accuracy of contemporary ECG interpretation criteria in risk stratification of master athletes

**DOI:** 10.3389/fcvm.2025.1637853

**Published:** 2025-09-02

**Authors:** Geza Halasz, Bruno Capelli, Simone Pasquale Crispino, Andrea Segreti, Raffaella Mistrulli, Federico Andreoli, Armando Ferrera, Francesco Perone, Michele Villa, Tiziano Cassina, Vincenzo Biasini, Francesco Grigioni, Domenico Gabrielli, Massimo Piepoli

**Affiliations:** ^1^ Cardiology Department, Azienda Ospedaliera San Camillo Forlanini, Rome, Italy; ^2^ Sport and Exercise Medicine, Istituto Cardiocentro Ticino, Ente Ospedaliero Cantonale, Lugano, Switzerland; ^3^ Department of Cardiovascular Sciences, Fondazione Policlinico Universitario Campus Bio-Medico di Roma, Rome, Italy; ^4^ Department of Movement, Human and Health Sciences, University of Rome “Foro Italico”, Rome, Italy; ^5^ Department of Clinical and Molecular Medicine, Sapienza University of Rome, Rome, Italy; ^6^ Cardiovascular Center Aalst, OLV Hospital, Aalst, Belgium; ^7^ Institute of Sports Medicine and Science, National Italian Olympic Committee, Rome, Italy; ^8^ Cardiac Rehabilitation Unit, Rehabilitation Clinic “Villa delle Magnolie”, Caserta, Italy; ^9^ Cardiac Anesthesia and Intensive Care, Istituto Cardiocentro Ticino, Ente Ospedaliero Cantonale, Lugano, Switzerland; ^10^ Italian Sport Medicine Federation Clinic, L’Aquila, Italy; ^11^ Clinical Cardiology, IRCCS Policlinico San Donato, Milan, Italy; ^12^ Department of Biomedical Science for Health, University of Milan, Milan, Italy

**Keywords:** master, preparticipation screening, ECG, sudden death, athlete

## Abstract

**Background:**

Limited data are available on the diagnostic performance of contemporary ECG interpretation criteria in master athletes (aged over 35 years). This study aimed to describe ECG findings and compare the diagnostic accuracy of the 2017 International, 2010 ESC, and 2013 Seattle criteria in identifying high-risk cardiovascular conditions in a cohort of competitive master athletes.

**Methods:**

We included 506 consecutive Caucasian master athletes (mean age, 47.9 ± 8.7 years; 85.6% male) who underwent ECG-based preparticipation screening. ECGs were retrospectively interpreted according to the three criteria. Transthoracic echocardiography was included to calculate sensitivity and specificity.

**Results:**

Thirteen athletes (2.5%) were diagnosed with a condition potentially related to sudden cardiac death (SCD), including severe aortic regurgitation (*n* = 1), Type 1 Brugada pattern (*n* = 1), chronic coronary syndromes (*n* = 4), dilated cardiomyopathy (DCM) (*n* = 3), aortic dilation (*n* = 3), and moderate aortic stenosis (*n* = 1). Diagnostic accuracy for conditions at risk of SCD was 0.73 for International, 0.81 for Seattle, and 0.77 for ESC criteria. Seattle demonstrated a significantly higher AUC than the International criteria (*p* = 0.0032). The International criteria failed to identify two athletes with DCM and left axis deviation, while no significant structural abnormalities were found in athletes with complete right bundle branch block (RBBB). The most common ECG abnormalities were left axis deviation (7.1%), left atrial enlargement (4.2%), and T-wave inversion (3%). A prolonged QT interval was diagnosed in 5.7% according to ESC criteria but in only one athlete under the International and Seattle criteria.

**Conclusion:**

The Seattle criteria demonstrated the highest overall accuracy, with significantly better discriminative performance than the International criteria, and a lower false-positive rate compared with the ESC criteria. These findings support the use of the Seattle criteria as part of a comprehensive screening strategy in master athletes.

## Introduction

The benefits of physical activity for cardiovascular health in aging populations are well-documented, with regular exercise linked to reduced risk of cardiovascular and metabolic diseases, including coronary artery disease (CAD), diabetes, hypertension, and obesity (1–4). Current guidelines recommend a minimum of 150 min of moderate or 75 min of vigorous exercise per week (1). However, master athletes—individuals aged 35 years and older who participate in competitive or high-intensity sports and often exceed these activity guidelines—constitute a unique group demonstrating distinct cardiovascular adaptations and risks due to prolonged, intense training (2, 5, 6). Master athletes have become increasingly common over the past few decades as sports such as marathons, triathlons, and cycling grow in popularity among older adults (7). While the health benefits of exercise are substantial, the potential adverse effects of high-intensity, high-volume training in master athletes have come under scrutiny (8). Some evidence suggests that long-term intense physical activity in this group may increase the risk of atrial fibrillation, CAD with elevated coronary artery calcification, unexplained myocardial fibrosis, and ventricular arrhythmias (3, 4, 9). Unlike younger athletes, where sudden cardiac death (SCD) is often associated with inherited or congenital heart disorders, SCD in master athletes is more commonly due to acquired conditions such as CAD (3, 4).

This complex risk profile highlights the importance of thorough cardiovascular screening in master athletes, aiming to balance the benefits of exercise with the risk of adverse cardiovascular events.

In this regard, electrocardiography (ECG) is widely recommended for preparticipation screening (PPS) by major sports cardiology and sports medicine societies to identify cardiovascular abnormalities that could predispose athletes to SCD (10, 11).

To improve the accuracy of the PPS, the criteria for interpreting the athlete's ECG have been revised several times (12–14).

These modifications aim to minimize false-positive results and increase the sensitivity in identifying athletes who are at risk of SCD.

However, while a significant amount of evidence exists on the interpretation of young adult athletes’ ECG (16–35 years) and our group has previously analyzed pediatric athletes (15, 16), data from the interpretation of ECG in master athletes are lacking and mainly derived from small and retrospective studies (17).

The aim of the present study was (i) to characterize the main electrocardiographic features of a cohort of master athletes aged over 35 years competing at regional and national levels in various sports disciplines and (ii) to evaluate and compare the diagnostic accuracy of three contemporary ECG interpretation criteria, the 2010 European Society of Cardiology recommendations (2010 ESC), the 2013 Seattle criteria, and the 2017 International recommendations, in identifying cardiovascular conditions associated with sudden cardiac death in this population.

## Methods

This study was conducted retrospectively on consecutive master athletes (aged over 35 years old) who presented for PPS at the University of L’Aquila (L’Aquila, Italy) and Cardiocentro Ticino (Lugano, Switzerland) between January 2018 and December 2020. Both male and female athletes were included, while those with a documented history of cardiovascular conditions linked to SCD or prior screenings at other institutions were excluded.

### General schedule

**Figure 1:**
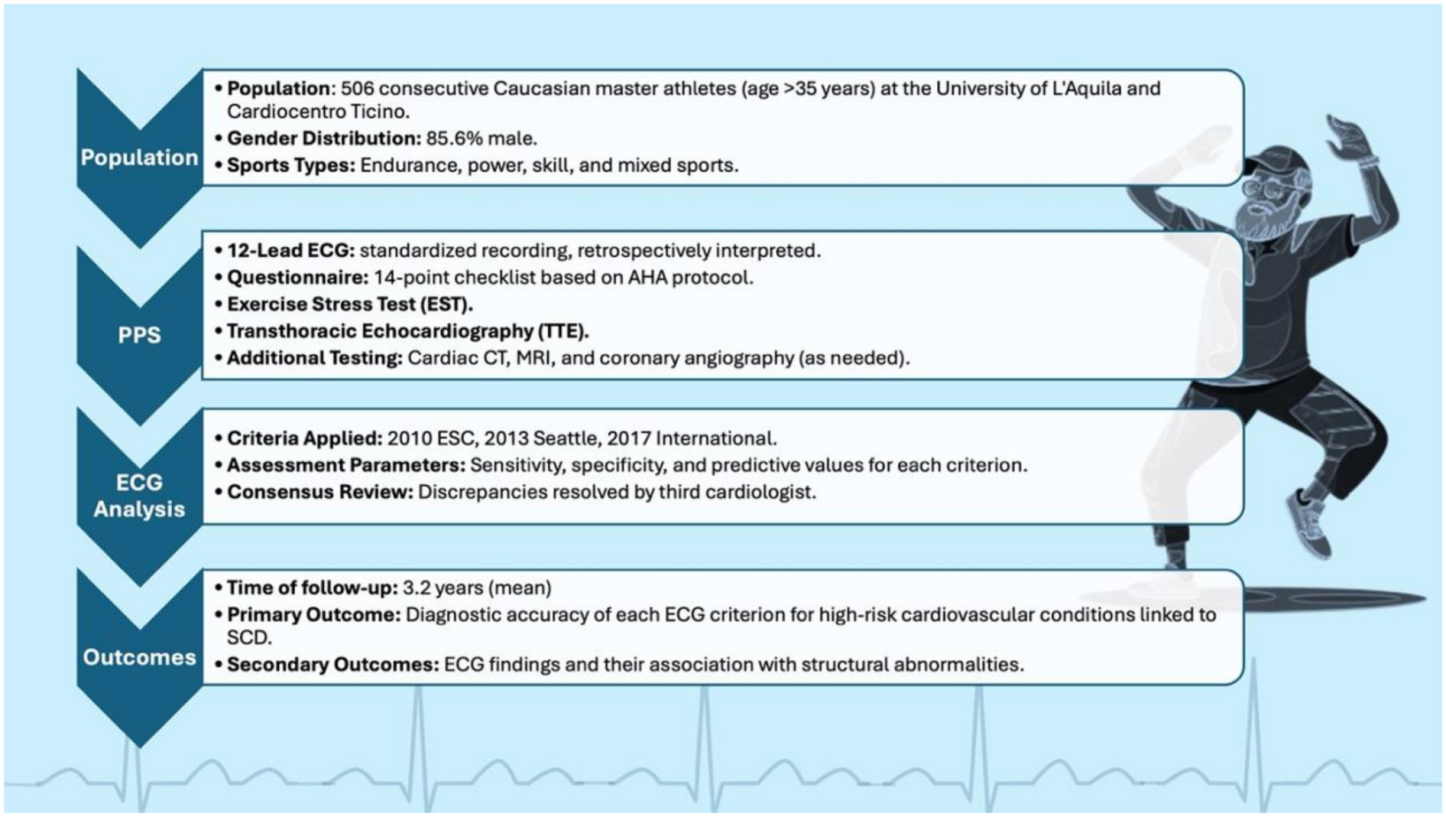
Study design and flowchart of master athletes undergoing preparticipation cardiovascular screening. A total of 506 consecutive athletes over 35 years of age were evaluated with a standardized protocol including questionnaire, 12-lead ECG, exercise stress testing, and transthoracic echocardiography. ECGs were retrospectively interpreted according to the 2010 ESC, 2013 Seattle, and 2017 International criteria, with diagnostic accuracy assessed during a mean 3.2-year follow-up for conditions associated with sudden cardiac death.

Sports were categorized into four groups according to the type of training-induced cardiovascular adaptation (TRCA), namely, power, endurance, skill, and mixed, according to the 2020 ESC Sports Cardiology guidelines (1). Each athlete underwent a comprehensive PPS protocol, including a self-reported questionnaire based on a 14-point checklist from the American Heart Association (18), a 12-lead resting ECG, an exercise stress test (EST), and transthoracic echocardiography (TTE). The need for additional, second-level investigations—such as cardiac computed tomography (CT), cardiac magnetic resonance imaging (MRI), or coronary angiography—was determined by the screening physicians, following national and international guidelines based on findings from the initial screening tests (Figure 1).

### Electrocardiogram and transthoracic echocardiography

ECGs were recorded both at rest and during EST using a standard 12-lead configuration with CardioSoft (GE HealthCare, Milwaukee, WI, USA) and Quark C12x (COSMED, Albano Laziale, Rome, Italy) systems. The ECG interpretations were conducted retrospectively by two independent sports medicine physicians (GH and BC), who were blinded to patient history and physical examination data. ECGs were analyzed according to the 2010 ESC, 2013 Seattle criteria, and 2017 International, with any discrepancies resolved through consensus with a third blinded cardiologist (SR). Measurements included heart rate, P-wave duration, PR interval, QRS duration, QT interval (in lead II), and R and S amplitudes, with QT intervals corrected for heart rate using Bazett's formula.

Definitions for ECG abnormalities included early repolarization (elevation of the QRS–ST junction by ≥0.1 mV, often with a QRS slurring or notching) and T-wave inversion (TWI) of ≥1 mm in ≥2 contiguous leads, excluding leads III, V1, and aVR. TWI was further categorized by lead location (anterior, inferior, lateral, and inferolateral). Juvenile T-wave patterns (anterior TWI in athletes <16 years old) were considered normal. EST was conducted in accordance with the protocol of the Italian Federation of Sports Medicine and the American Society of Sports Medicine (19). To enhance clarity, a comparative summary of the main ECG interpretation criteria is provided in Table 1.

**Table 1:** Comparative summary of the 2010 ESC, 2013 Seattle, and 2017 International ECG interpretation criteria, highlighting definitions of normal, borderline, and abnormal findings in athletes.

Category	ECG finding	2020 ESC	2017 International	2013 Seattle
Normal findings	Early repolarization	Considered normal	Considered normal	Considered normal
	Sinus bradycardia	Normal	Normal	Normal
	Incomplete RBBB	Considered normal	Considered normal	Considered abnormal
	First-degree AV block (PR ≤ 400 ms)	Normal	Same	Same
	Isolated QRS voltage for LVH	Considered normal	Considered normal	Considered normal
Borderline findings	Left axis deviation (less than −30°)	Borderline (acceptable if isolated)	Not formally defined	Not defined
	Right axis deviation (greater than +120°)	Borderline (acceptable if isolated)	Not formally defined	Not defined
	Atrial enlargement	Borderline (acceptable if isolated)	Not formally defined	Not defined
Abnormal findings	QTc prolongation (>470 ms men/>480 ms women)	Abnormal	Abnormal	Abnormal
	T-wave inversion (TWI) beyond V2 (white athletes)/beyond V4 (black athletes)	Abnormal	Similar but criteria less strict	Any TWI considered suspicious
	Ventricular pre-excitation	Abnormal	Abnormal	Abnormal
	Pathological Q waves	Abnormal (duration >40 ms or depth ≥25% R wave)	Same	Same
	ST-segment depression	Abnormal	Abnormal	Abnormal

TTE was performed using commercially available systems including the Vivid i (GE HealthCare, Milwaukee, WI, USA), E-Cube 7 (Alpinion Medical Systems, Seoul, Korea), and CX50 (Philips, USA). All TTEs were conducted by two experienced sports medicine physicians (GH and VB), with disagreements reviewed by a cardiologist (SR). Diagnostic criteria for hypertrophic cardiomyopathy (HCM), dilated cardiomyopathy (DCM), and arrhythmogenic cardiomyopathy (AC) followed current guidelines (20).

### Follow-up

In compliance with the requirements of the Italian Federation of Sport Medicine (FMSI) for annual PPS, all athletes were followed up on yearly from January 2018 until December 2020. New cardiovascular diagnoses and relevant clinical and diagnostic information—including data from ECG, EST, and TTE—were systematically documented in an electronic health record (EHR) to minimize bias and confounding factors.

### Statistical analysis

The normality of continuous variables was assessed using the Kolmogorov–Smirnov test. Categorical variables were expressed as counts and percentages, while continuous variables with a normal distribution were reported as means ± standard deviation (SD). Sensitivity, specificity, and positive and negative predictive values for the three ECG criteria were calculated using the conventional 2 × 2 contingency table approach to evaluate each criterion's effectiveness in identifying athletes at risk of SCD due to electrical or structural cardiac abnormalities. Comprehensive analyses incorporated all available diagnostic tests to confirm final diagnoses. A *p*-value of <0.05 was considered statistically significant, and all analyses were performed using SPSS version 21.0 (SPSS Inc., Chicago, IL, USA).

To compare the diagnostic performance of the three ECG interpretation criteria, we conducted both pairwise and global statistical analyses. The McNemar test was used to assess differences in paired proportions of sensitivity and specificity between criteria, based on matched binary classifications (true positives/negatives). This test evaluates whether the proportion of discordant classifications (e.g., cases classified as abnormal by one criterion but not the other) differs significantly.

In addition, we applied the DeLong test for correlated ROC curves to compare the area under the curve (AUC) values across the three criteria, as all were applied to the same cohort. This non-parametric method accounts for the correlated nature of AUCs derived from identical subjects and allows formal testing of differences in overall discriminative accuracy. A *p*-value <0.05 was considered statistically significant for both tests.

## Results

### Study sample

The study included 506 consecutive Caucasian athletes with a mean age of 47.9 ± 8.4 years, of whom 85.6% were male. Athletes participated in 17 different sports, with an average training duration of 8 ± 3.5 h per week. These sports were categorized into four groups based on TRCA, with endurance sports being the most common (65.8%), followed by mixed (23.1%), power (8.9%), and skill-based sports (2.2%). Most athletes were asymptomatic and presented no familiarity with cadiovascular disease (98.8%), while six athletes (1.2%) reported a family history of SCD. At the physical examination, 30 athletes (5.9%) were found to have blood pressure >140/90 mmHg, and 6 athletes (1%) had a pathological heart murmur. Thirteen athletes (2.5%) were diagnosed with conditions potentially related to SCD, including severe aortic regurgitation (*n* = 1), Type 1 Brugada syndrome (*n* = 1), chronic coronary syndromes (*n* = 4), dilated cardiomyopathy (*n* = 3), aortic dilation (*n* = 3), and moderate aortic stenosis (*n* = 1).

### ECG abnormalities

The frequency and type of ECG abnormalities varied according to the ECG criteria applied. The most common abnormalities identified were left axis deviation (7.1%), left atrial enlargement (4.2%), and T-wave inversion (3%). Notably, 29 athletes (5.7%) exhibited a prolonged QT interval according to the 2010 ESC criteria, compared with only 1 athlete identified by both the 2017 International and 2013 Seattle criteria. Of these athletes, 5 (1%) also showed abnormal findings on physical examination, while 10 (2%) had abnormal ECGs according to both the 2010 ESC and 2013 Seattle criteria and 6 (1.2%) according to the 2017 International criteria. Interobserver reliability for categorizing ECGs as abnormal was very good across all criteria, with kappa values of 0.88 (95% CI: 0.74–0.96) for 2010 ESC, 0.92 (95% CI: 0.84–0.94) for 2013 Seattle, and 0.91 (95% CI: 0.89–0.92) for 2017 International. Table 2 presents the main characteristics of the study population.

**Table 2:** Demographic and clinical characteristics of the athletes.

Factors	Master (*N* = 506)	Mean ± SD or *n* (%)	Seattle
Demographic characteristics			
Age, years	47.9	±8.4	
Male gender	433	(85.6)	
Height, cm	175	±75	
Weight, kg	75	±11	
BMI, kg/m^2^	24.6	±2.8	
SBP	128	±9	
DBP	77	±7	
Symptoms	6	(1.2)	
Pre-syncope	1	(0.2)	
Syncope	1	(0.2)	
Palpitations	3	(0.6)	
Chest pain	1	(0.2)	
Physical examination abnormalities	40	(7.9)	
Heart murmurs ≥ 2/6	5	(1.0)	
Split second heart sound	1	(0.2)	
Pectus excavatum	4	(0.8)	
Hypertension	30	(5.9)	
Family history of CVD	6	(1.2)	
Transthoracic echocardiography			
IVS (mm)	10.6	±1.3	
PWD (mm)	10.4	±1.3	
EDD (mm)	50.0	±5.1	
ECG parameters			
HR, bpm	69	±12	
P-wave duration	104	±16.1	
PR interval	165	±25.8	
QRS duration	92.8	±11.3	
Sokolow index	32.1	±9.1	
QRS limb voltage	14.1	±6.8	
QTc interval	420	±18.8	
Maximal HR	159	±13	
Recovery HR (3 min)	88	±16	
Recovery QTc	421	±25	
QTc interval stratification			
QTc ≥480 ms (F); QTc ≥470 ms (M)	1	(0.2)	According to the 2017 International criteria
QTc >460 ms (F); QTc >440 ms (M)	29	(5.7)	According to the 2010 ESC criteria
QTc <320 ms	0	(0.0)	
QTc <380 ms	9	(1.8)	
Sport classified according to cardiac adaptation to exercise			
Endurance	333	(65.8)	
Skill	11	(2.2)	
Power	45	(8.9)	
Mixed	117	(23.1)	

AV, atrioventricular; CVD, cardiovascular disease; BMI, body mass index; EDD, end-diastolic diameter; DBP, diastolic blood pressure; HR, heart rate; IVSd, interventricular septum; LVH, left ventricular hypertrophy; QTc, corrected QT interval; PWD, posterior wall; RBBB, right bundle branch block; SCD, sudden cardiac death; SBP, systolic blood pressure; SD, standard deviation; Mean ± SD or *n* (%); F, female; M, male.

### Findings from exercise stress testing, Holter monitoring, and echocardiography

Among the 506 athletes, 4 with normal resting ECGs demonstrated exercise-induced ST-segment changes suggestive of myocardial ischemia. All underwent coronary computed tomography angiography (CCTA), which excluded obstructive coronary artery disease in every case. No athlete experienced sustained ventricular arrhythmias during the exercise stress test.

In addition, 11 athletes underwent 24-h Holter ECG monitoring due to the presence of frequent premature ventricular contractions (PVCs) during the exercise test. None of these athletes showed evidence of atrial fibrillation or sustained ventricular arrhythmias, and no new structural or electrical diagnoses emerged in this subgroup.

Transthoracic echocardiography identified structural alterations in 16 athletes (3.2%) that warranted clinical follow-up. These included:

• mild aortic regurgitation in 7 athletes,
• mitral valve prolapse with mild regurgitation in 4 athletes (all with negative Holter and stress testing),
• mild left ventricular hypertrophy [mean interventricular septum (IVSd) 12.7 mm] in 2 athletes,
• mild systolic dysfunction (LVEF ∼53%) in 1 athlete without arrhythmias or late gadolinium enhancement on cardiac MRI, and
• patent foramen ovale with mild right-to-left shunt in 2 athletes.

### Follow-up

During a mean follow-up period of 3.2 years (range, 1–4 years), athletes continued to train without experiencing adverse cardiovascular events such as cardiac arrest, syncope, exertional symptoms, impaired exercise performance, or new structural cardiac disease. Three athletes diagnosed with chronic coronary syndrome underwent further evaluation with coronary angiography. Of these, two athletes with normal left ventricular function returned to competitive sports after successful revascularization, while one athlete with incomplete revascularization was disqualified from competitive participation. Three athletes with aortic dilation but without presenting features of Marfan syndrome, Loeys–Dietz syndrome, or familial thoracic aortic aneurysm were monitored with echocardiography and limited to low- and moderate-intensity sports. The three athletes with dilated cardiomyopathy were referred to a tertiary center for a comprehensive evaluation; one tested negative for genetic mutations, while two tested positive for laminin mutations. The athlete with moderate aortic stenosis was allowed to participate only in low-intensity sports, while the athlete with severe aortic regurgitation underwent surgical valve replacement (Table 3).

**Table 3:** Patients diagnosed with abnormalities associated with sudden cardiac death.

Age	Sex	Sport	Exam with abnormalities	Second test	Diagnosis	ECG findings	2017 International	2010 ESC	2013 Seattle
44	M	Cycling	TTE	CT	Aortic dilation	Positive Sokolow's criteria (46 mm)	Normal	Normal	Normal
51	M	Track and field	TTE	CT	Aortic dilation	Normal	Normal	Normal	Normal
65	M	Track and field	ECG	CT	Aortic dilation	Left atrial enlargement	Normal	Pathological	Pathological
49	M	Cycling	ECG	TTE CMR 24h-AECG EST	DCM	Left axis deviation	Normal	Pathological	Pathological
37	M	Track and field	ECG	TTE CMR 24h-AECG EST	DCM	Left axis deviation	Normal	Pathological	Pathological
70	M	Track and field	ECG	TTE CMR 24h-AECG EST	DCM	Left Anterior fascicular block	Normal	Pathological	Pathological
48	M	Cycling	ECG	TTE 24h-AECG	Type 1 Brugada	Type 1 Brugada	Pathological	Pathological	Pathological
65	M	Cycling	ECG	TTE Coronary angiography	Chronic coronary syndromes	Left fascicular block	Pathological	Pathological	Pathological
60	M	Track and field	ECG	TTE CT Coronary angiography	Chronic coronary syndromes	Lateral T-wave inversion	Pathological	Pathological	Pathological
61	M	Track and field	ECG clinical examination	TTE CT Coronary angiography	Chronic coronary syndromes, hypertension	Lateral T-wave inversion	Pathological	Pathological	pathological
54	M	Cycling	Clinical examination ECG	TTE CT Coronary Angiography	Chronic coronary syndromes, hypertension	Left axis deviation	Pathological	Pathological	Pathological
51	M	Soccer	Clinical examination	TTE	Moderate aortic stenosis	Lateral T-wave inversion	Pathological	Pathological	Pathological
72	M	Tennis	Clinical examination	TTE TEE	Severe aortic regurgitation	Positive Sokolow's criteria (44 mm)	Normal	Normal	Normal

CMR, cardiac magnetic resonance imaging; ECG, electrocardiography; EPS, electrophysiology studies; EST, exercise stress test; F, female; LV, left ventricular; M, male; SAECG, signal-averaged electrocardiography; TTE, transthoracic echocardiography; 24h-AECG, 24-h ambulatory ECG monitoring; IRBB, incomplete right bundle brunch block; CT, computed tomography; PJRT, permanent junctional reciprocating tachycardia; ERP, early repolarization; AC, arrhythmogenic cardiomyopathy.

### Diagnostic accuracy of ECG criteria

We assessed the diagnostic accuracy of the three ECG criteria for detecting conditions associated with SCD using several metrics.

The 2017 International criteria showed an accuracy of 0.73, with a sensitivity of 0.62 (95% CI: 0.32–0.86) and a specificity of 0.85 (95% CI: 0.81–0.88).

**Figure 2:**
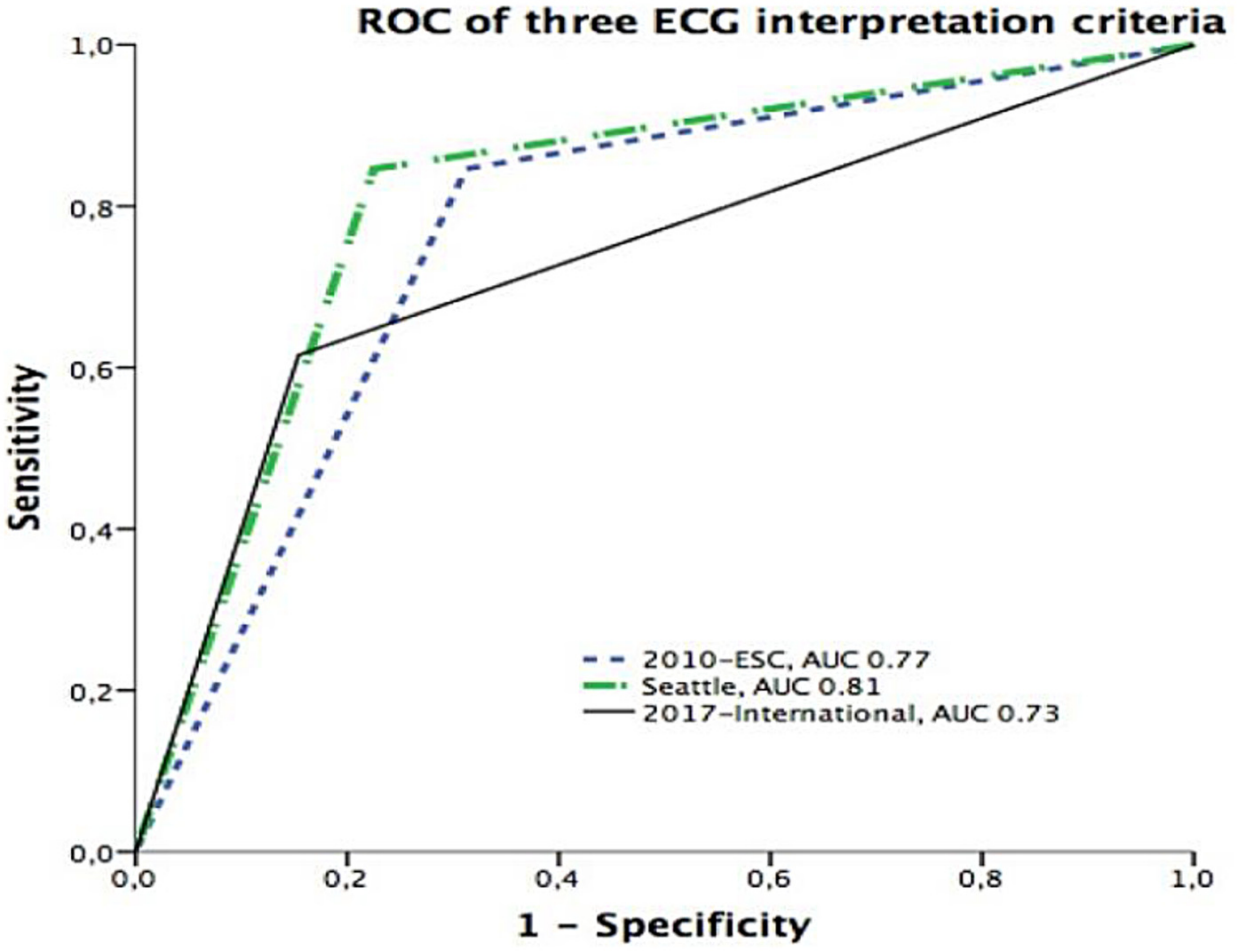
Receiver operating characteristic (ROC) curves comparing the predictive performance of three ECG interpretation criteria. The Seattle criteria demonstrated the highest discriminative power with an area under the curve (AUC) of 0.81, followed by the 2010 ESC criteria (AUC = 0.77) and the 2017 International recommendations (AUC = 0.73).

The 2010 ESC and 2013 Seattle criteria both had a sensitivity of 0.85 (95% CI: 0.55–0.98) but differed in specificity, with values of 0.69 (95% CI: 0.64–0.73) for ESC and 0.77 (95% CI: 0.74–0.81) for Seattle (Figure 2). The false-positive rate was highest for the ESC criteria (30.4%), followed by the Seattle (21.9%) and International criteria (15%). The false-negative rate was lowest with the ESC and Seattle criteria (0.4%) compared with the International criteria (1%).

Further significant values included the positive predictive value (PPV) and negative predictive value (NPV) for each criterion. The PPVs were 0.10 (International), 0.07 (ESC), and 0.09 (Seattle), while NPVs were uniformly high at 0.99 across all criteria, indicating strong reliability in excluding high-risk conditions. The positive likelihood ratio (PLR) was highest for the International criteria (3.99) compared with Seattle (3.8) and ESC (2.7), while the negative likelihood ratio (NLR) was lowest for Seattle (0.20) and ESC (0.22) compared with International (0.45), further supporting the balance achieved by the Seattle criteria. The area under the curve (AUC) values reflected overall diagnostic accuracy, with the highest AUC observed for Seattle (0.81), followed by ESC (0.77) and International (0.73).

Findings from the direct comparison of each ECG criteria are summarized in Table 4.

**Table 4:** Diagnostic performance of the three ECG interpretation criteria in master athletes for pathologies at risk of SCD.

Parameter	Master (>35 years) (*N* = 506)		
	2017 International	2010 ESC	2013 Seattle
Sensitivity	0.62 (0.32–0.86)	0.85 (0.55–0.98)	0.85 (0.55–0.98)
Specificity	0.85 (0.81–0.88)	0.69 (0.64–0.73)	0.77 (0.74–0.81)
False-positive rate	15	30.4	21.9
False-negative rate	1	0.4	0.4
Positive predictive value	0.10 (0.06–0.15)	0.07 (0.05–0.09)	0.09 (0.07–0.12)
Negative predictive value	0.99 (0.98–0.99)	0.99 (0.98–1.0)	0.99 (0.98–1.0)
Positive likelihood ratio	3.99 (2.48–6.4)	2.7 (2.0–3.5)	3.8 (2.83–4.99)
Negative likelihood ratio	0.45 (0.23–0.91)	0.22 (0.06–0.80)	0.20 (0.05–0.71)
Area under the curve (AUC)	0.73 (0.69–0.77)	0.77 (0.73–0.80)	0.81 (0.77–0.84)

Sudden cardiac death (SCD)-associated conditions are structural or electrical cardiac disorders that confer an increased risk of life-threatening ventricular arrhythmias, particularly during physical exertion.

Values are reported as proportions (%) with 95% confidence intervals (CI).

Seattle vs. International *p* = 0.0032; Seattle vs. ESC *p* = 0.11; ESC vs. International *p* = 0.14.

### Comparative diagnostic performance

The McNemar paired test showed no significant difference in combined sensitivity and specificity between Seattle and International (*χ*² = 0.57, *p* = 0.45) or ESC and International (*χ*² = 0.57, *p* = 0.45).

In contrast, the DeLong test indicated that the AUC for the Seattle criteria (0.81, 95% CI: 0.77–0.84) was significantly higher than that of the International criteria (0.73, 95% CI: 0.69–0.77; *p* = 0.0032). Differences in AUC between Seattle and ESC (0.77, 95% CI: 0.73–0.80; *p* = 0.11) and between ESC and International (*p* = 0.14) did not reach statistical significance.

## Discussion

The findings from this study underscore the importance of selecting appropriate ECG interpretation criteria for preparticipation screening (PPS) in master athletes. Our results revealed significant differences in the sensitivity and specificity of the 2010 ESC, 2013 Seattle, and 2017 International ECG criteria for detecting cardiovascular conditions associated with SCD.

The Seattle criteria demonstrated the best balance, with high sensitivity (85%) and specificity (77%), while the International criteria, despite achieving the highest specificity (85%), had lower sensitivity (62%). The ESC criteria also had high sensitivity (85%) but suffered from lower specificity (69%), resulting in a higher rate of false positives. Importantly, the difference in AUC between the Seattle and International criteria was statistically significant (*p* = 0.0032), while the differences between Seattle and ESC, and between ESC and International, did not reach statistical significance.

These variations in diagnostic performance, although modest, are crucial, as they directly influence clinical decision-making and the management of master athletes in practice.

While our study focused on the comparative diagnostic performance of different ECG interpretation criteria, we fully acknowledge the need for a multimodal, individualized approach in master athletes, as supported by the 2020 ESC Sports Cardiology Guidelines and studies such as the MASS trial. Master athletes represent a unique population due to the combined effects of age-related cardiovascular changes and training-induced adaptations.

In our cohort, six individuals (1.2%) reported a positive family history (PFH) of cardiovascular disease (CVD), as shown in Table 2. None of these athletes were diagnosed with conditions associated with sudden cardiac death (SCD) during screening or follow-up.

While PFH is recognized as a red flag in young athletes—particularly for inherited cardiomyopathies and channelopathies—its clinical impact in master athletes is less pronounced, as the risk profile shifts toward acquired diseases (e.g., coronary artery disease). Nonetheless, PFH remains relevant and should prompt more detailed history taking, potentially including a three-generation pedigree and clarification of the exact nature, age, and cause of cardiac events in relatives.

For athletes with PFH, especially if accompanied by symptoms or borderline ECG findings, longitudinal follow-up is essential, even in the absence of initial abnormalities.

Age-related changes, such as increased atrial size, mild left ventricular hypertrophy, and altered repolarization patterns, can resemble pathologic findings, leading to potential misinterpretation of ECG (4).

This is particularly relevant in older athletes, where conditions such as left axis deviation, left atrial enlargement, and right bundle branch block (RBBB) are commonly encountered but may not always signify pathology (21).

In our study, the high specificity of the International criteria, while beneficial in reducing false positives, was associated with lower sensitivity, which resulted in missed diagnoses of conditions such as dilated cardiomyopathy (DCM) and chronic coronary syndromes.

These findings echo those of other studies that have found lower sensitivity to be a limitation of the International criteria in older athletes (21). Clinically, the use of criteria with lower sensitivity may lead to underdiagnosis of critical conditions, potentially leading to adverse cardiovascular events during competition. For master athletes, where acquired cardiovascular diseases such as CAD are more prevalent, the ability of the screening criteria to accurately detect these conditions is essential.

False positives are another major consideration in ECG-based PPS, as they can lead to unnecessary follow-ups, increased anxiety for the athlete, and a higher burden on healthcare systems (22).

The ESC criteria, while sensitive, were associated with the highest false-positive rate (30.4%). This aligns with existing literature that notes a tendency for the ESC criteria to overestimate abnormalities, particularly in older athletes (23).

From a clinical perspective, excessive false positives can lead to additional testing, which is costly and may not be easily accessible in all healthcare settings. In contrast, the Seattle criteria showed a lower false-positive rate (21.9%), supporting their utility in reducing unnecessary follow-ups while still maintaining high sensitivity. The practical impact of this balance translates to fewer referrals for advanced imaging, reducing both healthcare costs and the psychological burden on athletes.

The detected interobserver reliability of all three criteria was very good, suggesting that the criteria themselves are robust and reproducible across practitioners, regardless of which criteria are chosen. This high interobserver reliability is an important finding, as it supports the use of ECG as a standard tool in PPS, providing consistent results across different evaluators.

However, the criteria's inherent limitations, particularly in sensitivity and specificity, underscore the need for a multimodal approach to screening in master athletes.

The authors believe that incorporating advanced imaging techniques when needed, only in doubtful cases, such as cardiac magnetic resonance imaging (MRI) and coronary computed tomography angiography (CCTA), could enhance the accuracy of PPS by identifying structural and coronary abnormalities that may not be evident on ECG alone.

Kramer et al. (24) highlighted the potential of these imaging modalities, in particular in the detection and management of myocardial fibrosis and coronary calcifications in asymptomatic athletes, underscoring their value in comprehensive cardiovascular risk assessment.

One key critical finding of our study was the 2017 International criteria's failure to identify two athletes with DCM and left axis deviation on ECG, which underscores a potential and dangerous limitation of these criteria set in detecting certain pathologies since they are correlated with worse prognosis in the long-term.

Different from the pediatric counterpart (15), in our cohort, all athletes with RBBB were found to have no significant structural abnormalities, suggesting that this ECG finding may be considered benign in asymptomatic master athletes.

This study has several strengths that enhance its relevance in assessing cardiovascular risk in master athletes. First, the focus on master athletes addresses a gap in cardiovascular research for older, physically active individuals, with findings that carry practical implications for sports cardiology and PPS protocols. Furthermore, it provides a direct comparison of three widely recognized ECG interpretation criteria—the 2010 ESC, 2013 Seattle, and 2017 International guidelines—focusing on their diagnostic accuracy for detecting conditions associated with SCD in athletes over 35, which was not performed in the past in this category of patients. This comparative approach is particularly valuable, as it highlights the strengths and limitations of each criterion within the unique physiological profile of aging athletes, offering insights that may guide the selection of the most appropriate criteria for PPS in this demographic. The study's comprehensive PPS protocol, including 12-lead ECG, EST, and TTE, adds rigor to the diagnostic assessment by allowing for a comprehensive evaluation of each criterion's sensitivity and specificity.

The inclusion of TTE, which provides structural cardiac data to corroborate ECG findings, further strengthens the accuracy of diagnostic conclusions.

Nonetheless, the study presents limitations.

The relatively low number of athletes diagnosed with conditions associated with sudden cardiac death limits the statistical power of comparative analyses, particularly for AUC-based performance comparisons. While the sample size of 506 provides valuable real-world insight into a screening population of master athletes, we acknowledge that the study may be underpowered to detect small-to-moderate differences between ECG interpretation criteria with sufficient confidence. Future studies with larger event numbers will be needed to validate and refine these comparative findings. Additionally, the relatively low proportion of female athletes may have limited, at least in part, the evaluation of sex-specific differences in ECG patterns, particularly regarding T-wave inversion, which could represent a potential source of variability in ECG interpretation. Selection bias may also be present, as master athletes at competitive levels may self-select out or be excluded by previous screenings, potentially underestimating cardiovascular risk. A small proportion of athletes in our cohort reported mild symptoms or a family history of SCD. While this may modestly affect prevalence estimates, we intentionally included these individuals to reflect real-world screening practice in master athletes. Unlike studies on younger, low-risk populations, our cohort represents the heterogeneity typically encountered in clinical settings. This choice enhances external validity but may limit direct comparison with previous validation studies of ECG criteria.

Moreover, variability in cardiovascular adaptation across sports disciplines introduces heterogeneity that may influence the diagnostic performance of ECG criteria. Finally, retrospective ECG interpretation, despite blinding, carries an inherent risk of bias.

## Conclusions

This study provides a comparative evaluation of three widely used ECG interpretation criteria.

The 2013 Seattle criteria emerged as the most balanced, with high sensitivity and specificity, offering a reliable tool for PPS in master athletes.

Clinically, these findings highlight the need for tailored PPS protocols in master athletes, integrating ECG criteria with an optimal balance between sensitivity and specificity. Future research involving larger, more diverse cohorts and prospective designs is required to validate these findings, refine ECG criteria for master athletes, and ensure alignment of screening protocols with the unique cardiovascular profiles of this population.

This approach would aim to support safe participation in competitive sports while reducing the psychological and healthcare impacts of false-positive results.

## Data Availability

The raw data supporting the conclusions of this article will be made available by the authors, without undue reservation.

## In memoriam

The authors acknowledge the valuable contribution of VB to this study and dedicate the manuscript to his memory.
